# Eosinophilic Myocarditis Demonstrated Using Cardiac Magnetic Resonance Imaging in a Patient with Eosinophilic Granulomatosis with Polyangiitis (Churg-Strauss Disease)

**DOI:** 10.7759/cureus.2792

**Published:** 2018-06-12

**Authors:** Tarun Dalia, Sonya Parashar, Nilay V Patel, Archana Gautam, Hongyan Dai, Steven Bormann

**Affiliations:** 1 Internal Medicine, University of Kansas, Kansas City, USA; 2 Department of Cardiology, University of Kansas, Kansas City, USA; 3 Internal Medicine, UAB School of Medicine,montgomery, Montgomery, USA; 4 Pathology, University of Kansas, Kansas city, USA; 5 Department of Cardiology, University of Kansa, Kansas City, USA

**Keywords:** eosinophilic myocarditis, egpa, churg strauss disease, cardiac magnetic resonance imaging, five factor score, elevated troponins, coronary cta

## Abstract

Eosinophilic granulomatosis with polyangiitis (EGPA), historically known as the Churg-Strauss disease, is a small- to medium-sized vessel multi-organ vasculitis with a propensity to involve the heart. EGPA is a rare condition with an estimated annual incidence of one to 4.2 people per million. The cardiac involvement causes significant morbidity and mortality in EGPA patients. Approximately 50% of the deaths in EGPA are related to cardiac disease and occur within the first few months since diagnosis. The current recommendations support evaluation of cardiac involvement by using history, physical exam and multimodality imaging including echocardiogram and cardiac magnetic resonance imaging (CMR). Here, we report a rare case of eosinophilic myocarditis in a 19-year-old patient with EGPA seen on CMR. Pertinent literature is also reviewed. We highlighted the importance of CMR in diagnosing and follow up of EGPA patients.

## Introduction

Eosinophilic granulomatosis with polyangiitis (EGPA) formerly known as Churg-Strauss Syndrome, was first described in 1951 as a form of disseminated necrotizing vasculitis with extravascular granulomas in patients with underlying asthma and tissue eosinophilia [1-3]. EGPA is now classified as small- to medium-sized vessel vasculitis. It is distinguished from other forms of vasculitis with its strong association with asthma and eosinophilia [2,4,5]. EGPA is a rare condition with an estimated annual incidence of one to 4.2 people per million [6]. The mean age of onset is between 38 and 54 years with an equal preference between men and women [3]. Approximately 30-40% of EGPA patients are antineutrophil cytoplasmic antibody (ANCA)-positive and have an increased frequency of mononeuritis multiplex and glomerulonephritis whereas the ANCA-negative patients tend to be more prone to cardiomyopathy [4,7]. Cardiac involvement in EGPA can occur in about 60% of patients and can present as pericarditis, pericardial effusion, acute heart failure, acute myocardial infarction, valvular heart disease, and myocarditis. A 48% mortality rate is associated with cardiac involvement in EGPA patients [8-10]. There has been an increasing interest in the use of cardiac magnetic resonance imaging (MRI) in the evaluation and monitoring of EGPA patients although its clinical utility is still unclear [10-13]. Here we report a rare case of myocarditis in a 19-year-old patient who initially presented with fatigue and atrial fibrillation and was subsequently diagnosed with EGPA.

## Case presentation

An ill-appearing 19-year-old male with the one-year history of asthma presented to the emergency room with non-specific symptoms including fatigue, dyspnea, numbness in the right leg, nausea, vomiting, and dizziness. Two months prior to presentation, he had a sinus surgery and within few days after this surgery, he developed cough and dyspnea, so he was admitted to outside hospital for possible pneumonia. He was treated with cefuroxime, Tamiflu, and oral prednisone. He improved momentarily with steroids. Two weeks later, he returned to the outside hospital complaining of right foot plantar numbness and dyspnea, he was discharged home on Levaquin as they thought he may have some residual sinus disease left. One week later he was seen by a pulmonologist at outside hospital and they noticed that one of the cultures grew staph, hence started on Bactrim. He took Bactrim for three days and his mother noticed that he developed some mental status changes, hence Bactrim was stopped. After this, no more symptom of mental status change was noticed. Over the next few weeks, the patient noticed tachypalpitations, continued to have fatigue, shortness of air, and fatigue so the family decided to come to our hospital's emergency department for further workup. While in the emergency room, he was found to be in atrial fibrillation with the rapid ventricular response and elevated troponins. The patient spontaneously converted into sinus rhythm within 10 minutes. His vital signs were stable except for tachycardia with a heart rate of around 100 beats per minute. Physical examination was unremarkable with a normal sensation on right leg and foot. He was admitted to cardiac intensive care unit for further workup due to elevated troponin.

Salient laboratory values and electrocardiogram

**Figure 1:**
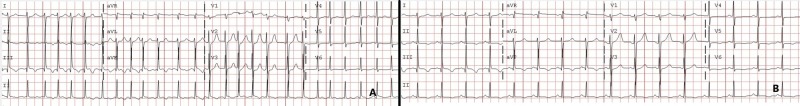
Electrocardiogram. (A) Initial electrocardiogram (ECG) showed atrial fibrillation with a heart rate of 161, non-diagnostic Q waves in the inferior leads, T-wave inversions in the inferior leads and no significant ST segment changes noted. (B) Repeat ECG 10 minutes later showed sinus tachycardia with a heart rate of 100, nondiagnostic Q waves in the inferior leads and T-wave inversions in the inferior leads as well and no significant ST segment changes noted.

The patient’s initial complete blood count was remarkable for white blood cell of 28,800/ul with eosinophil count of 12,960/ul (45%) in spite of the use of low-dose oral corticosteroids for a few days prior to admission. Erythrocyte sedimentation rate (ESR) and C-reactive protein (CRP) were both elevated at 35 and 4.14, respectively. His admission troponin was 16.28. His initial electrocardiogram (ECG) showed atrial fibrillation with a heart rate of 161 beats per minute, non-diagnostic Q waves in the inferior leads, T-wave inversions in the inferior leads and no significant ST segment changes noted (Figure 1A). His repeat ECG 10 minutes later when he converted to sinus rhythm showed sinus tachycardia with a heart rate of 100 beats per minute, Q and T changes as noted earlier, as well and no significant ST segment changes noted (Figure 1B). Other labs, including TSH, UDS, BNP, lactate, and renal function, were unremarkable. Rheumatological workup including anti-nuclear antibody (ANA), perinuclear antineutrophil cytoplasmic antibody (p-ANCA), cytoplasmic antineutrophil cytoplasmic antibody (c-ANCA), rheumatoid factor, myeloperoxidase (MPO) antibody, serine protease antibody 3, and anti-cyclic citrullinated peptide (anti-CCP) IgG was inconclusive. However, the patient’s IgE and IgG were both markedly elevated. Several infectious causes, such as histoplasma, coccidioides, strongyloides, cytomegalovirus (CMV), human immunodeficiency virus (HIV), tuberculosis (TB), Epstein-Barr virus (EBV), hepatitis B, and hepatitis C, were explored and all were negative.

Imaging

**Figure 2:**
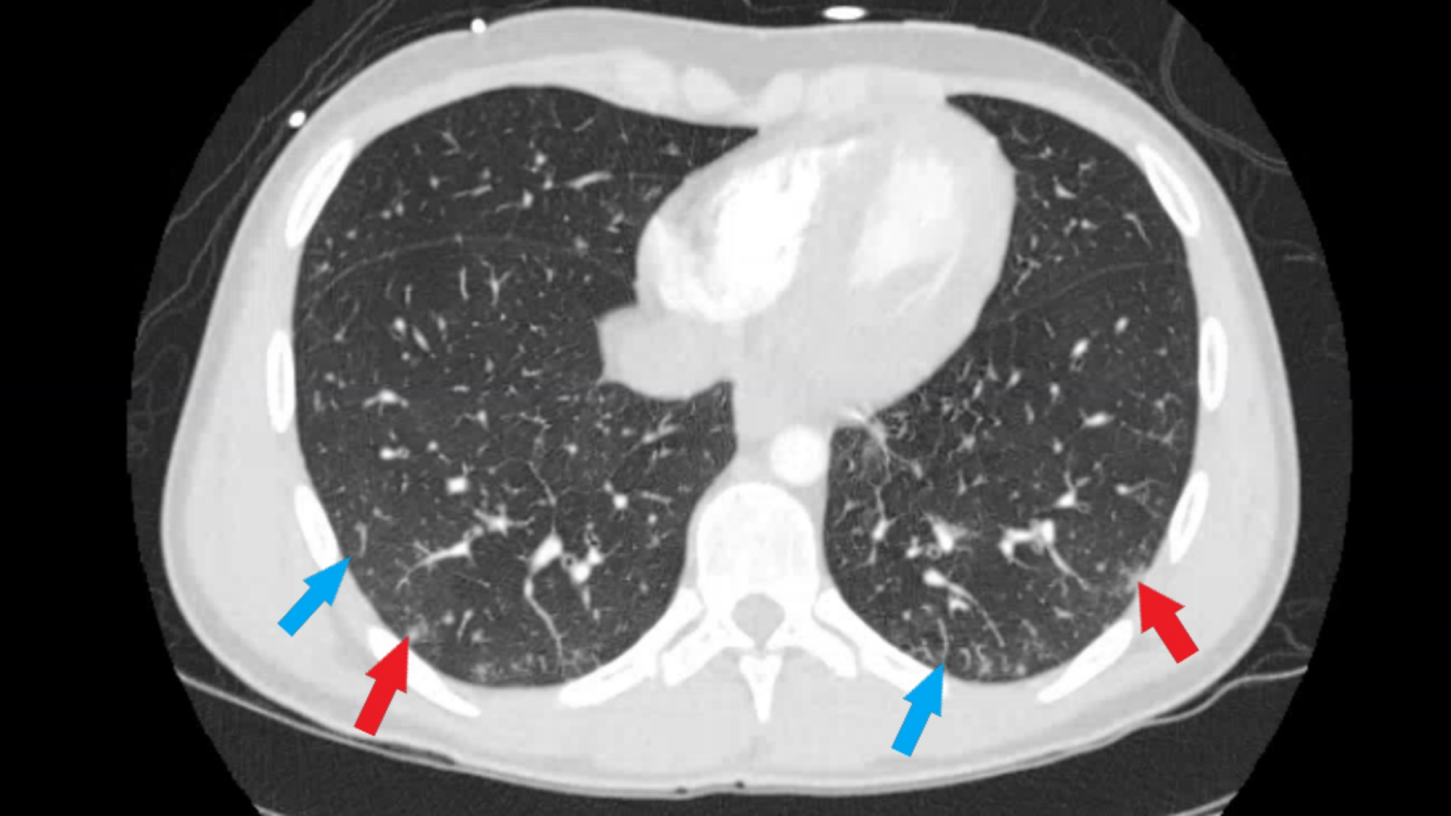
Computed tomography angiography (CTA) chest. CTA of the chest: Bilateral patchy ground-glass opacities (blue arrow), greatest at the posterior lower lobes. Some additional more nodular appearing opacities (red arrow) are present and also greater in the lower lobes.

**Figure 3:**
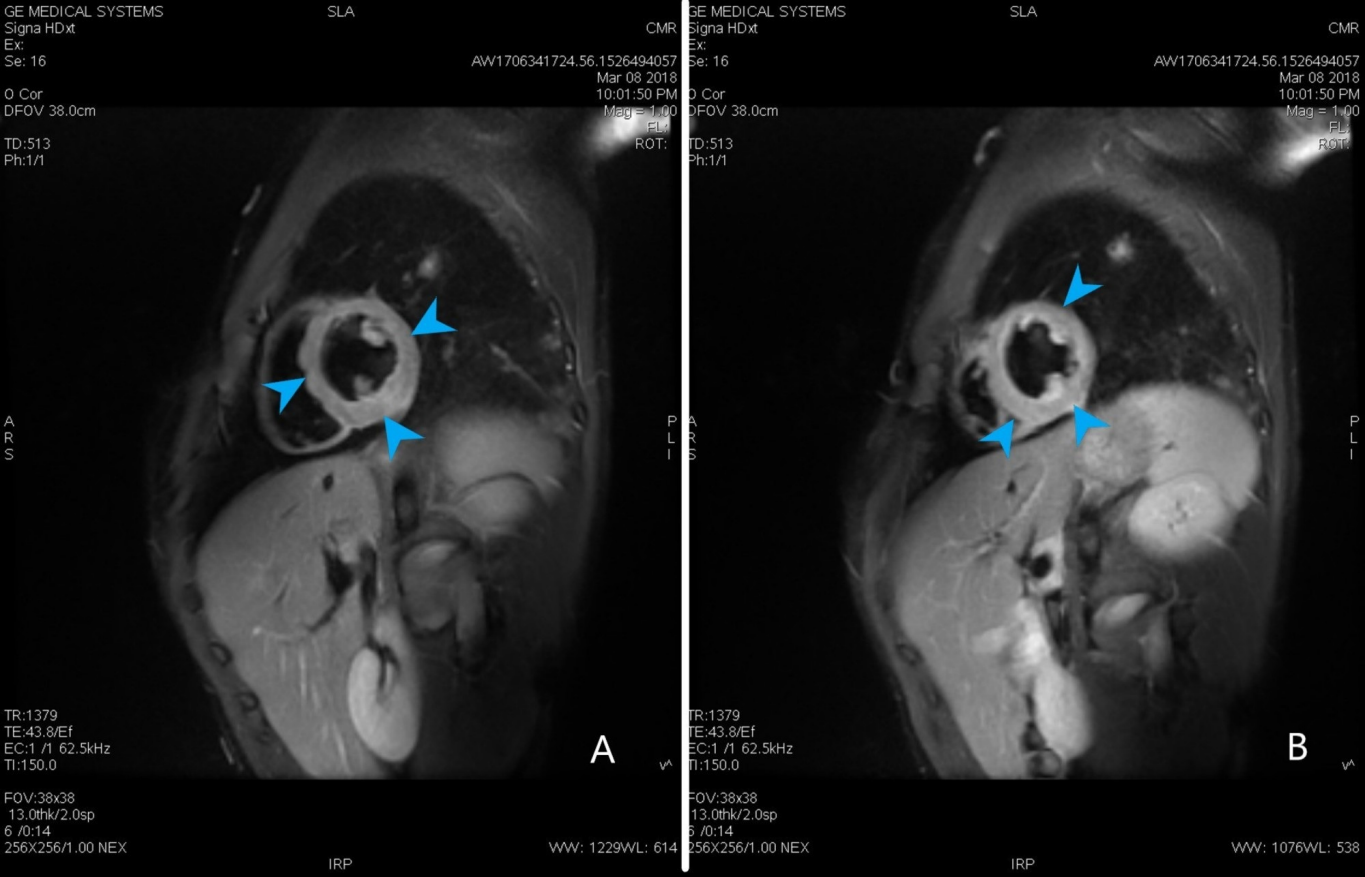
Cardiac magnetic resonance. (A, B) Cardiac magnetic resonance triple inversion T2 weighted image showing edema of the left ventricle wall (blue arrow heads).

**Figure 4:**
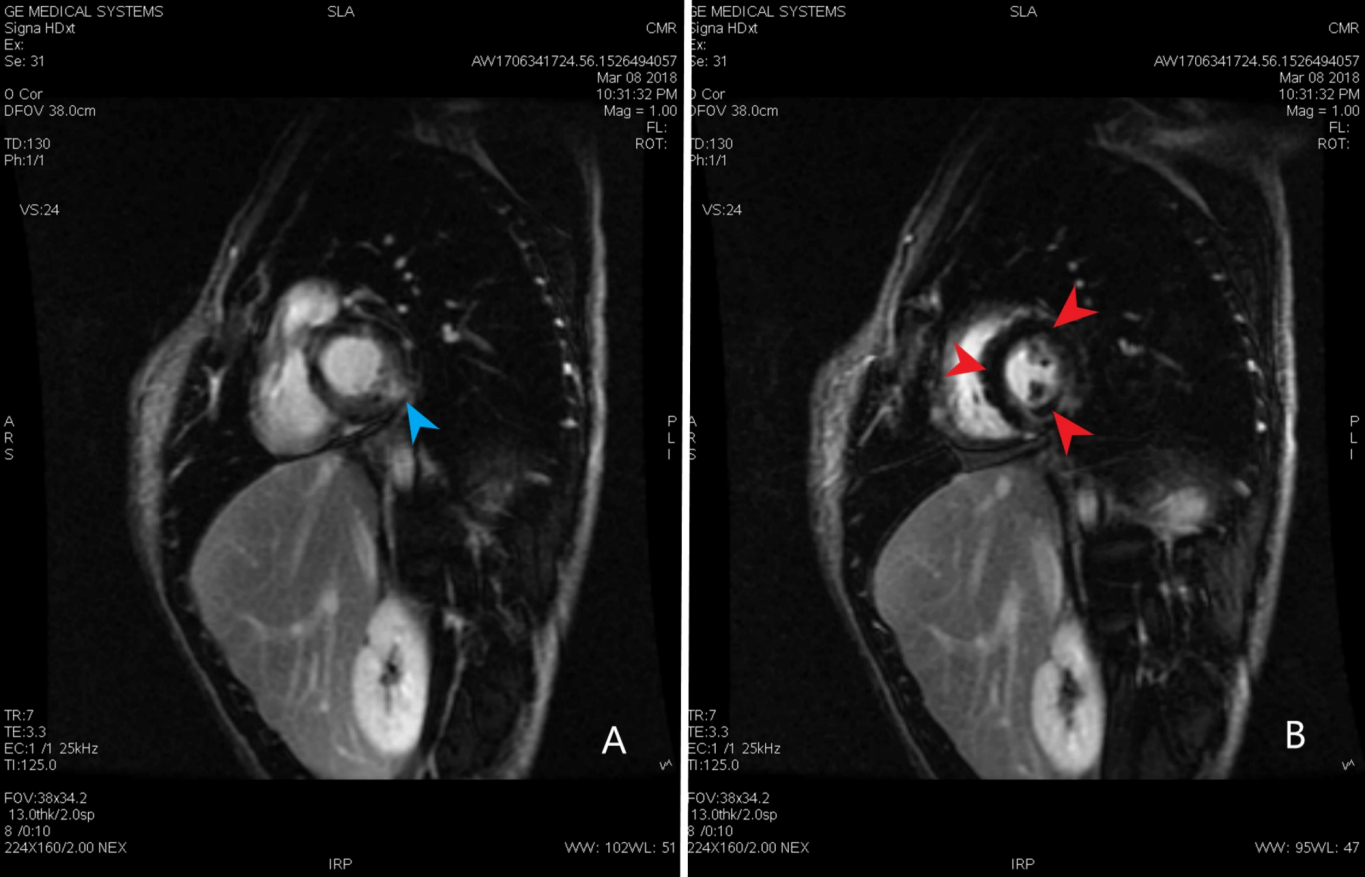
Cardiac magnetic resonance. (A, B) Cardiac magnetic resonance short axis view showing patchy areas of the delayed myocardial enhancement of the Gadolinium within the left ventricular myocardium (blue arrow head). The abnormal delayed myocardial enhancement pattern is most consistent with myocarditis. There is fairly discrete region of decreased perfusion in the mid to apical septal and inferior segments and throughout the apex (red arrow heads). Segmental perfusion abnormality on dynamic imaging may be the result of hypoperfusion associated with vasculitis.

Transthoracic echocardiogram revealed an ejection of 55% with some apical hypokinesis. The transesophageal echocardiogram showed no evidence of endocarditis, thrombus, shunt, or atherosclerosis. Computed tomography angiography (CTA) of the chest with and without contrast showed moderate mediastinal and bilateral hilar adenopathy in addition to bilateral axillary lymphadenopathy, bilateral peribronchial thickening, and patchy ground-glass opacities most predominantly in the posterior lower lobes. There was no evidence of pulmonary embolism (Figure 2). Cardiac magnetic resonance (CMR) showed several areas of delayed enhancement within the left ventricular myocardium and decreased perfusion in the mid to apical septal and inferior segments throughout the apex. It also revealed a small pericardial effusion and minimal hypokinesis of the lateral apical wall (Figures 3-4). Due to the abnormal myocardial enhancement, a CT of the heart with coronary CTA was ordered which showed normal coronary artery anatomy with no evidence of stenosis, calcified plaque, or soft plaque (Videos 1-4). Due to his reported neurologic symptoms, CT of the head without contrast was ordered and showed two areas of low-attenuation within right frontal white matter. MRI of the head was subsequently performed which showed many small bilateral punctate infarcts throughout cerebrum and a few additional ones in the cerebellum.

**Video 1:** Coronary CTA - LAD view. View of LAD coronary artery. Patent LAD. CTA: Computed tomography angiography; LAD: Left anterior descending.

**Video 2:** Coronary CTA - Diagonal artery. View of Diagonal artery (Branch of LAD). Patent Diagonal Artery. CTA: Computed tomography angiography; LAD: Left anterior descending.

**Video 3:** Coronary CTA- LCx view. Left circumflex view (LCx). Patent LCx CTA: Computed tomography angiography.

**Video 4:** Coronary CTA- RCA view. View of right coronary artery (RCA). Patent RCA. CTA: Computed tomography angiography.

Biopsies

**Figure 5:**
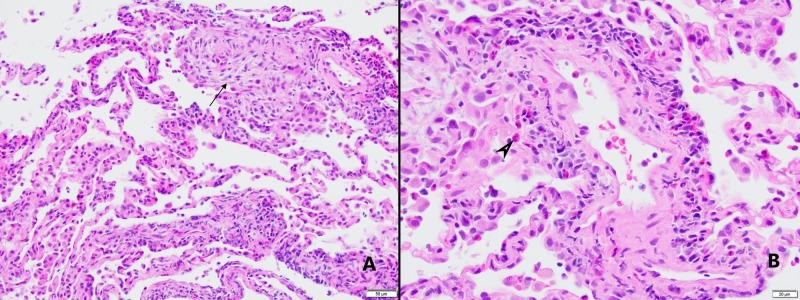
Pathology images of cryobiopsies. (A) 20x image. Microscopic examination demonstrates dense eosinophilic infiltrate involving alveolated lung parenchyma and many small venules, accompanied by patchy organization (black arrow) (H&E stained sections, 20x). (B) 40x image. Eosinophilic venulitis. Chronic inflammation including many eosinophils infiltrating venular wall (black arrow head) (H&E stained sections, 40x).

A bone marrow biopsy showed normocellular bone marrow for age and no concern for dysplasia; however, both the bone marrow biopsy and peripheral blood smear showed marked eosinophilia with leukocytosis. Several transbronchial cryobiopsies were taken from the left lower, upper lobes and lingula of the lung which showed patchy areas of eosinophilic venulitis with dense eosinophilic infiltrates involving many of the small venules. This process was happening in the background of chronic bronchiolitis with abundant eosinophils within small airways, smooth muscle hypertrophy, and goblet cell metaplasia (which suggests asthma). All these findings taken into consideration together suggested EGPA (Figure 5).

The patient was initially started on 1000 mg of intravenous methylprednisolone for three days and then 1 mg/kg/day of oral prednisone for several months with a gradual taper. He was also started on cyclophosphamide for three to six months. Additionally, due to the patient’s young age, arrangements for sperm preservation were made prior to starting cyclophosphamide. The patient responded well to the treatment and at his one-month rheumatology follow-up, the patient continued to improve. His troponin-I reduced to 0.08 at one month visit.

## Discussion

EGPA is an unusual disease which presents as systemic vasculitis and peripheral eosinophilia in patients with chronic atopic disease [14]. Hypereosinophilia and eosinophilic myocarditis can occur in a variety of settings including allergic diseases, drug reactions, parasitic infections, malignancies like lymphoma, systemic disorder like idiopathic hypereosinophilic syndromes, vasculitis and more [12].

Cardiac involvement in patients with EGPA varies widely from 16 to 92% and is more common in ANCA negative patients [7]. This is in concordance with our patient who also had ANCA negative eosinophilic myocarditis. EGPA is one of the most common types of the systemic vasculitides to affect the heart, which greatly increases morbidity and mortality. Approximately 50% of the deaths in EGPA are related to cardiac disease and occur within first few months since diagnosis [7]. When EGPA affects the heart, it can lead to a myriad of presentations like pericarditis, pericardial effusion, acute heart failure, acute myocardial infarction, valvular heart disease, and myocarditis [8-10]. Our patient presented with rhythm disturbances, namely atrial fibrillation and sinus tachycardia. Eosinophilic myocarditis was diagnosed based on his history of asthma, peripheral neuropathy, a very high peripheral eosinophilia, elevated troponins, ESR and CRP, rhythm disturbances and CMR showing several areas of delayed enhancement within the left ventricular myocardium. Other causes of peripheral eosinophilia including drug reaction, parasitic or other infections, and malignancy were ruled out. There are two main mechanisms of cardiac involvement in patients with EGPA: eosinophilic infiltration of myocardium and vasculitis-related ischemia [15].

The prognosis of EGPA is generally good in treated patients [4]. The Five Factor Score (FFS) is used to evaluate the prognosis of vasculitides at time of diagnosis. The revised 2009 FFS includes four factors for poorer prognosis and one factor for better outcome. The following four factors are associated with poor prognosis: age > 65 years, cardiac symptoms, gastrointestinal involvement and renal insufficiency (each factor is given +1 point). Ear, nose and throat symptoms in patients with Wegener granulomatosis or EGPA are associated with lower risk of death (their absence is given +1 point). According to the 2009 FFS for systemic necrotizing vasculitides, such as polyarteritis nodosa, microscopic polyangiitis, EGPA, the five-year mortality rate for scores of 0, 1 and ≥2 were 9%, 21%, and 40%, respectively [16]. The cytotoxic drugs are recommended with FFS ≥ 1 [13]. Corticosteroids and cyclophosphamide are classically used for induction, while azathioprine and methotrexate are the therapeutic options for maintenance [5]. In our patient, high dose corticosteroids in addition to cyclophosphamide were used for induction.

In many cases of the EGPA patients may not even have cardiac symptoms or ECG abnormalities. Therefore, it is recommended to evaluate cardiac involvement in these patients by detailed history, physical examination, ECG and multimodality imaging, such as an echocardiogram or CMR [17]. There are different ways to access for the cardiac involvement in EGPA like an echocardiogram, endomyocardial biopsy and CMR [6]. CMR is a very safe and sensitive modality. CMR has a sensitivity of 88% and specificity of 72%, reveals cardiac abnormalities in 62% of EGPA patients as compared to 50% by echocardiogram [6, 7]. The prevalence of myocardial damage as depicted by late gadolinium enhancement (LGE) imaging is frequent in EGPA disease [7]. Our patient’s CMR also showed delayed gadolinium enhancement within the left ventricular myocardium and minimal hypokinesis of the left lateral wall. The exact role of CMR in diagnosis, prognosis, and monitoring clinical response is not fully elucidated yet. Many small studies and case reports support the use of CMR in EGPA patients as it provides detailed information about the cardiac structure. CMR is a valuable tool for early diagnosis of cardiac involvement and to show improvement during follow up sessions [6, 9, 17-19].

## Conclusions

We present a rare case of eosinophilic myocarditis in a patient with EGPA. Cardiac involvement is common and markedly contributes to morbidity and mortality in EGPA. Expeditious evaluation of cardiac involvement in EGPA drastically improves outcomes. CMR not only provides a way to promptly assess cardiac involvement in EGPA patients but it also helps to risk stratify patients and guide therapy. Early detection and treatment can save patients from developing late complications of endomyocarditis. We suggest that further large-scale studies are needed to assess the role of CMR in EGPA patients.
